# A175 MACHINE LEARNING MODELLING OF MULTIMORBIDITY PATTERNS AND PREMATURE MORTALITY IN INFLAMMATORY BOWEL DISEASE

**DOI:** 10.1093/jcag/gwae059.175

**Published:** 2025-02-10

**Authors:** G Tennakoon, G Postill, V Harish, I Itanyi, F Tang, E Buajitti, E Kuenzig, L Rosella, E Benchimol

**Affiliations:** University of Toronto Temerty Faculty of Medicine, Toronto, ON, Canada; University of Toronto Dalla Lana School of Public Health, Toronto, ON, Canada; University of Toronto Dalla Lana School of Public Health, Toronto, ON, Canada; University of Toronto Dalla Lana School of Public Health, Toronto, ON, Canada; The Hospital for Sick Children, Toronto, ON, Canada; University of Toronto Dalla Lana School of Public Health, Toronto, ON, Canada; The Hospital for Sick Children, Toronto, ON, Canada; University of Toronto Dalla Lana School of Public Health, Toronto, ON, Canada; The Hospital for Sick Children Department of Paediatrics, Toronto, ON, Canada

## Abstract

**Background:**

Multimorbidity is the co-occurrence of two or more chronic conditions in one individual. It is associated with reduced quality of life, poorer disease outcomes, increased hospitalizations, and polypharmacy. Although linked to premature mortality in general populations, the relationship between multimorbidity and premature mortality in inflammatory bowel disease (IBD) is unknown.

**Aims:**

(1) characterize multimorbidity patterns across the life course within the context of IBD; (2) identify how the sequence of condition accumulation contributes to premature death.

**Methods:**

We conducted a population-based retrospective matched cohort study using health administrative data from Ontario, Canada, including decedents with IBD between 2010 and 2020. Consensus K-Means Clustering was employed to characterize multimorbidity patterns, allowing us to identify potential groups of patients with similar condition profiles. Recurrent Neural Network (RNN) and Long Short-Term Memory (LSTM) models were trained to analyze the impact of the sequence of condition accumulation on premature mortality, considering either early-onset (diagnosed before 60 years of age) or all life conditions. These models were chosen for their ability to process sequential data, which is crucial for understanding how the order and timing of condition onset in multimorbidity progression impacts premature mortality.

**Results:**

Among 9,278 IBD decedents (49% female), we identified three multimorbidity clusters: (α) mood disorders and/or osteo- and other arthritis, (β) cancer with low multimorbidity, and (γ) cardiovascular comorbidities (Figure 1). Premature deaths accounted for 47.2% (n=4,380) of all deaths. All models performed well, with Area Under the Curve (AUC) values ranging from 0.84 to 0.88. The LSTM model achieved the best performance (AUC 0.88) in predicting premature mortality, using data from early-onset conditions. Key predictors of premature mortality were young ages of diagnosis for mood disorder, osteoarthritis, other mental health disorders, hypertension, and male sex.

**Conclusions:**

This study provides novel insights into multimorbidity patterns in IBD. Our findings reveal three specific multimorbidity clusters in the IBD population and we identified conditions important for predicting premature mortality. These results highlight the importance of providing multidisciplinary care for patients with IBD throughout their lives.

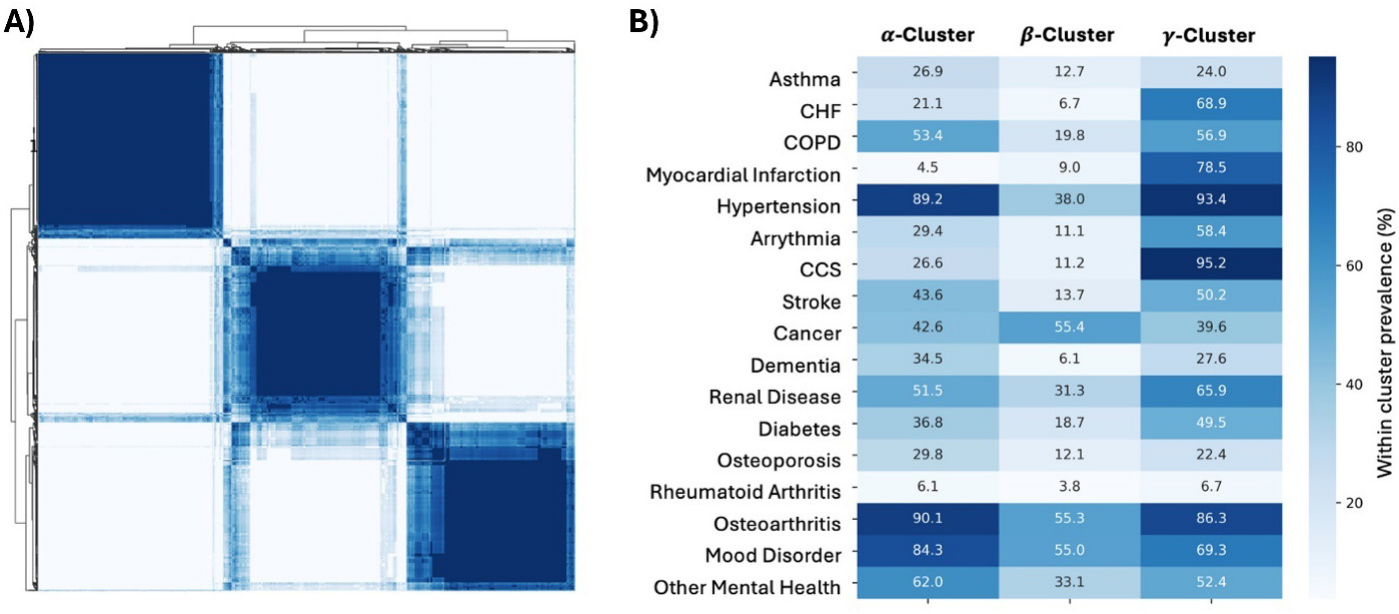

**Figure 1.** IBD patient multimorbidity clusters. (A) Consensus matrix showing three patient clusters based on comorbidities. Darker blue indicates more consistent patient pairing. (B) Heatmap showing chronic condition prevalence in the three clusters (α, β, γ). Darker blue indicates higher prevalence.

**Funding Agencies:**

None

